# The Intestinal Barrier Function and Intra-Abdominal Pressure Depend on Postoperative Analgesia Technique in Children with Appendicular Peritonitis

**DOI:** 10.1155/2021/6650361

**Published:** 2021-08-07

**Authors:** Valentyna Perova-Sharonova, Ulbolhan Fesenko

**Affiliations:** ^1^Department of Anaesthesiology and Intensive Care, Lviv Regional Pediatric Hospital, Lviv 79000, Ukraine; ^2^Department of Anaesthesiology and Intensive Care, Danylo Halytsky Lviv National Medical University, Lviv 79010, Ukraine

## Abstract

**Introduction:**

Peritonitis is one of the risk factors for the development of intra-abdominal hypertension (IAH) and abdominal compartment syndrome (ACS). The plasma citrulline and intestinal fatty acid-binding protein (I-FABP) are informative markers of intestinal barrier function. The aim of this study was to determine the correlation of the plasma citrulline and I-FABP with intra-abdominal pressure (IAP) and their relation to analgesia techniques in children suffering from appendicular peritonitis.

**Materials and Methods:**

74 children operated for appendicular peritonitis were randomized into three groups of postoperative analgesia: “Opioids” (*n* = 25), intravenous morphine of 10 mcg/kg/h; “Lidocaine” (*n* = 23), intravenous lidocaine with initial bolus of 1.5 mg/kg and then infusion of 1.5 mg/kg/h; and “EA” (*n* = 26), epidurally 0.25% bupivacaine with initial bolus of 1 mg/kg and then infusion of 0.4 mg/kg/year. Retrospectively patients in each group were divided into the following subgroups: “without IAH” (*n* = 33), “IAH” (*n* = 27), and “ACS” (*n* = 14). We detected citrulline and I-FABP in plasma on day 1 (D1) and day 3 (D3) of hospital stay.

**Results:**

The patients without IAH on D1 presented significantly higher plasma citrulline (23.7 (16.0–31.3) nmol/ml) and lower I-FABP (76.9 (32.6–121.1) pg/ml) levels compared with patients in subgroup “IAH” (9.3 (7.3–11.3) nmol/ml and 226.0 (161.8–290.3) pg/ml, respectively) and subgroup “ACS” (6.9 (5.3–8.6) nmol/ml and 1011.7 (731.9–1291.5) pg/ml, respectively). The IAP had strong inverse correlation (*r*_*s*_ = −0.74; *p* < 0.00001) with citrulline and positive strong correlation (*r*_*s*_ = 0.73; *p* < 0.00001) with I-FABP. The citrulline in patients with IAH during three days postoperatively increased significantly in “Lidocaine” to 72% (*p*=0.01) and in “EA” to 138% (*p*=0.02), but it decreased to 13% (*p*=0.37) in “Opioids” group. In children with ACS, citrulline on D3 was significantly higher than that on D1 and increased in “Lidocaine” to 59% (*p*=0.05) and in “EA” to 134% (*p*=0.001), but in “Opioids” it decreased to 30% (*p*=0.48). The I-FABP in patients with IAH decreased to 12% in “Lidocaine” group (*p*=0.86) and to 75% in “EA” group (*p*=0.01), but it increased to 37% (*p*=0.57) in “Opioids” group. During observation period, I-FABP in patients with ACS decreased significantly in “Lidocaine” to 42% (*p*=0.05) and in “EA” to 96% (*p*=0.003), but it increased in “Opioids” to 63% (*p*=0.22).

**Conclusions:**

The IAP was inversely correlated with plasma citrulline and positively correlated with I-FABP in children with appendicular peritonitis. Epidural analgesia is the most protective for intestinal wall barrier function in patients at risk of IAH and ACS.

## 1. Introduction

The WSACS (World Society of Abdominal Compartment Syndrome) defines the intra-abdominal hypertension (IAH) in children as a persistent or repeated increase in intra-abdominal pressure (IAP) above 10 mmHg and abdominal compartment syndrome (ACS) as the intra-abdominal hypertension accompanied by the manifestation of new organ dysfunction or deterioration of the existing one [1].

Peritonitis is one of the risk factors for the development of IAH and ACS due to intestinal paresis, edema of intestinal wall, and accumulation of effusion in the abdominal cavity [2]. The incidence rate of IAH and ACS among children with peritonitis was not defined. However, according to Basu et al. [3], IAH developed in 41% of adults with secondary peritonitis.

Disruption of the intestinal wall barrier is also associated with a poor prognosis in critically ill patients. Identification of specific biomarkers may be useful for early diagnosis of enterocyte damage. Decreased plasma concentrations of citrulline and increased I-FABP are common in critically ill patients with splanchnic hypoperfusion. These sensitive markers of intestinal wall insufficiency can detect intestinal damage before histological changes occur [4].

Adequate analgesia is highly recommended in the conservative therapy of IAH/ACS for improving the compliance of the abdominal wall, especially in postoperative period [1].

There are insufficient scientific data on the dependence of citrulline and I-FABP levels on the IAP values and postoperative analgesia technique in children with IAH.

The aim of this study was to determine the correlation of the enteral barrier function markers (citrulline and I-FABP) with intra-abdominal pressure and their relation to analgesia techniques in children suffering from appendicular peritonitis.

## 2. Materials and Methods

A positive conclusion on compliance with the principles of the Declaration of Helsinki, the Council of Europe Convention on Human Rights and Biomedicine, ICH GCP, and relevant laws of Ukraine was received from the Commission on Bioethics of Danylo Halytsky Lviv National Medical University (Protocol no. 1, January 31, 2018, Chairman Prof. AY Nakonechny).

The observational prospective study included 74 children with 105 (63; 135) months of age who underwent surgery for appendicular peritonitis at Lviv Regional Pediatric Clinical Hospital from February 2018 to February 2020. The informed consent to participate in the study was received prior to inclusion in the study from patients above 14 years of age and the parents of all patients.

The inclusion criteria were the following: age of 1–18 years, early postoperative stage after surgery for appendicular peritonitis, and absence of contraindications for intra-abdominal pressure measurement throw urinary bladder catheterization. The noninclusion criteria were the following: refusal of the patient or parents to participate in the study; anamnesis of inflammatory bowel diseases, congenital pathology with malabsorption, and surgical interventions for intestinal resection, which may affect citrulline and I-FABP levels; and the presence of contraindications to intra-abdominal pressure measurement throw urinary bladder catheterization.

After surgery, children were randomized into three groups depending on the method of postoperative analgesia, using a random number generator (https://www.random.org). The groups were comparable by clinical, anthropometric, and demographic data (see Table 1), as well as by types of surgery (see Table 2).

Postoperatively the analgesia protocols were the following:

- In patients of the “Opioids” group (*n* = 25), an intravenous infusion of morphine of 10 mcg/kg/h was administered
- Children of the “Lidocaine” group (*n* = 23) were given intravenous lidocaine with an initial bolus of 1.5 mg/kg and subsequent infusion of 1.5 mg/kg/h
- In children of the “EA” group (*n* = 26), epidural catheter was placed throw puncture site in Th12-L1 level using “loss of resistance” method and the catheter was advanced up to Th7-Th8 level; 0.25% bupivacaine was administered with an initial bolus of 1 mg/kg and subsequent infusion of 0.4 mg/kg/year

All children received intravenous paracetamol (60 mg/kg/day) as multimodal analgesia regimen.

The pain intensity was assessed at rest and during movements using numerical rating scales (NRS) in children above 7 years of age and Face, Legs, Activity, Cry, Consolability (FLACC) scale in those below 7 years of age. In cases of pain intensity ≥ 4 points, intravenous bolus of morphine was administered in a dose of 100 mcg/kg.

The intra-abdominal pressure was measured throw the urinary Foley catheter 4 times a day postoperatively according to the WSACS recommendations [1].

Retrospectively patients in each group were divided into subgroups according to the intra-abdominal pressure level and presence of organ dysfunction: “without IAH” (intra-abdominal pressure ˂ 10 mmHg), “IAH” (intra-abdominal pressure > 10 mmHg detected two or more times during study period), and “ACS” (abdominal compartment syndrome with signs of organ dysfunction).

The nasogastric tube was placed in all children for decompression. The conservative treatment was used in patients with IAH and ACS according to the WSACS algorithms [1]. Fluid therapy was given according to routine physiological requirements and volume of pathological fluid loss but avoiding big positive fluid balance. In cases of intestinal paresis on the third day after surgery, neostigmine (0.1 mg/kg) was used for stimulation of peristalsis.

Diazepam (2.5–5 mg) was used for sedation in patients with IAH/ACS for increasing the compliance of abdominal wall. The 14 patients with ACS were given respiratory support in the PSIMV mode with lung-protective strategy. 10 patients needed hemodynamic support with dobutamine of 5 mcg/kg/min, and one patient was given epinephrine of 0.05 mcg/kg/min. Decompressive laparotomy was performed in only one patient with ACS, and this case was lethal due to multiorgan dysfunction.

The blood samples were taken for determination of citrulline and I-FABP on the first day (D1) of hospital stay, immediately after surgery ended, and before the postoperative analgesia started and on the third day (D3) of hospital stay. The blood samples were taken using the vacutainers impregnated with heparin for citrulline and with EDTA for I-FABP in total amount of 4 ml. The samples were centrifuged for 15 min at 1000 rpm. The selected and labeled plasma was then frozen and stored at −20°C until analysis. Plasma citrulline and I-FABP levels were determined using highly specific, commercial kits for enzyme-linked immunosorbent assay (ELISA): Human Citrulline ELISA Kit (Cat No. MBS2601236; MyBioSource, Inc., San Diego, California, USA) and Human FABP2/I-FABP ELISA Kit (Cat No. MBS178728; MyBioSource, Inc., San Diego, California, USA) in automatic analyzer for microplates (ELx800™; BioTek Instruments, USA) according to the manufacturer's instructions.

### 2.1. Statistical Analysis

Data were analyzed using STATISTICA 8.0 (StatSoft Inc., USA). All data were presented as mean (95% CI). Statistical significance of differences was assessed using Student's *t*-test for data with normal distribution and Mann–Whitney's *U* test for data with nonnormal distribution. Correlations between variables were calculated using Spearman's correlation analysis and expressed as Spearman's correlation coefficient (*r*_*s*_). Differences and correlations were considered significant if *p* value was lower than 0.05. Graphics were made with mean and 95% confidence interval.

## 3. Results

All children were given an adequate analgesia. The average pain intensity at rest and during movements during three days postoperatively (see Table 3) was slightly higher in “Opioids” group than in “Lidocaine” (*p* < 0.001) and “EA” (*p* < 0.001) groups. There were no statistically significant differences between “Lidocaine” and “EA” groups in pain intensity (*p* > 0.05) as well as in morphine dose (*p* > 0.05).

Among all patients (*n* = 74), on day 1, IAH developed in 27 (36.5%) and ACS was diagnosed in 14 (19%) children. The patients without IAH presented significantly higher plasma citrulline and lower I-FABP levels compared with patients with IAH. In patients with IAH, plasma citrulline was significantly higher and I-FABP was significantly lower compared to patients with ACS (see Table 4).

**Figure 1:**
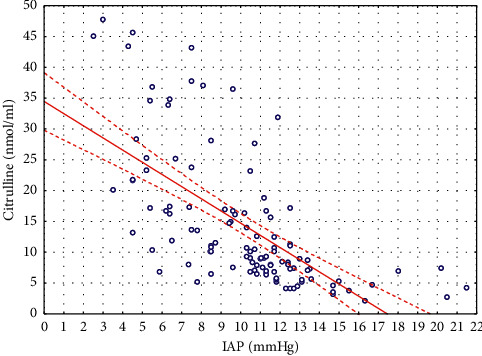
Correlations between IAP and plasma citrulline levels.

The IAP levels among all patients had statistically significant strong inverse correlation (*r*_*s*_ = -0.74; *p* < 0.00001) with plasma citrulline (Figure 1).

**Figure 2:**
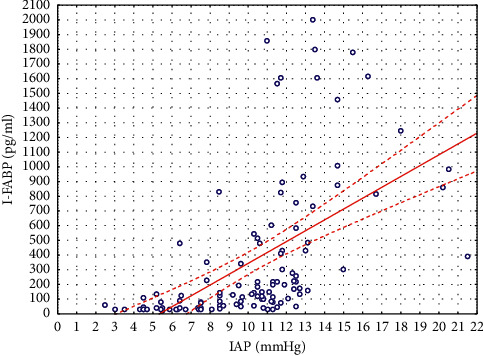
Correlations between IAP and plasma I-FABP levels.

**Figure 3:**
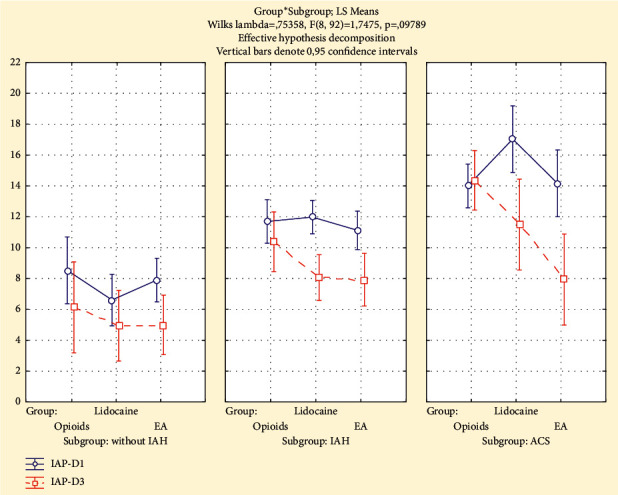
The intra-abdominal pressure (mmHg) on day 1 (IAP-D1) and day 3 (IAP-D3) in analgesia groups and subgroups (without IAH, IAH, and ACS).

Also a positive strong, statistically significant correlation (*r*_*s*_ = 0.73; *p* < 0.00001) was observed between the IAP levels and the plasma I-FABP among all children (Figure 2). (Figure 3)

### 3.1. The Dynamics of Intra-Abdominal Pressure Levels (Figure 3)

The average baseline IAP levels on day 1 (D1) among patients without IAH did not differ significantly between subgroups according to analgesia technique. The IAP decreased approximately to 2 mmHg during the observation period from day 1 (D1) to day 3 (D3) in all three groups.

Among the patients with IAH, the lowering of IAP during observation period was statistically significant in the “Lidocaine” (*p*=0.01) and “EA” (*p*=0.03) groups but not in the “Opioids” (*p*=0.11) group. In children with IAH in the “Lidocaine” and “EA” groups on the third day (D3) from the onset of analgesia, IAP values were significantly lower than those in the “Opioids” group (*p* < 0.05).

**Figure 4:**
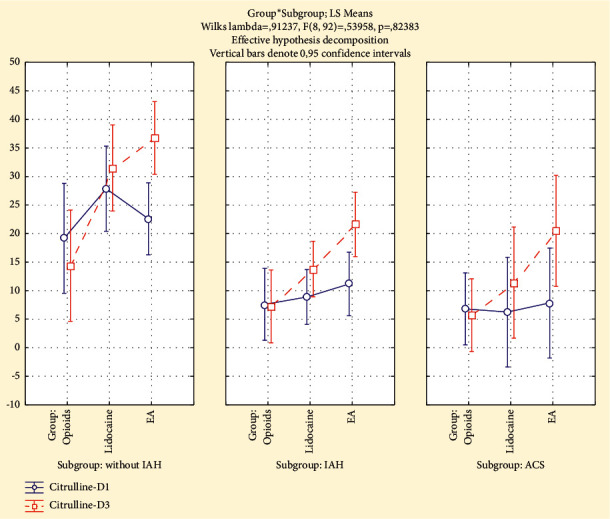
The plasma citrulline (nmol/ml) on day 1 (IAP-D1) and day 3 (IAP-D3) in analgesia groups and subgroups (without IAH, IAH, and ACS).

In patients with ACS, the average IAP was unchanged during the study period for three days postoperatively in the “Opioids” (*p*=0.86) group, but it decreased significantly in the “Lidocaine” group to 32% (*p*=0.05) and in the “EA” group to 48% (*p*=0.05). In children with abdominal compartment syndrome, in the “EA” group, IAP on the third day (D3) was significantly lower than that in the “Lidocaine” and “Opioids” groups (*p* < 0.05). (Figure 4)

### 3.2. The Dynamics of Plasma Citrulline

Among the patients without IAH, the plasma citrulline level during study period was almost unchanged in the “Opioids” and “Lidocaine” groups but not in the “EA” group where the plasma citrulline increased to 66% compared to initial level on D1 and it was significantly higher than that in the “Opioids” group on D3 (*p* < 0.05).

The average plasma citrulline levels among the patients with IAH during the study period decreased insignificantly to 13% (*p*=0.37) in the “Opioids” group and increased significantly in the “Lidocaine” group to 72% (*p*=0.01) and in the “EA” group to 138% (*p*=0.02). In children with IAH in the “Lidocaine” and “EA” groups on the third day (D3) from the onset of analgesia, plasma citrulline levels were significantly higher than those in the “Opioids” group (*p* < 0.05). Also, in the “EA” group on the third day (D3), the citrulline level was significantly higher than that in the “Lidocaine” group (*p* < 0.005).

**Figure 5:**
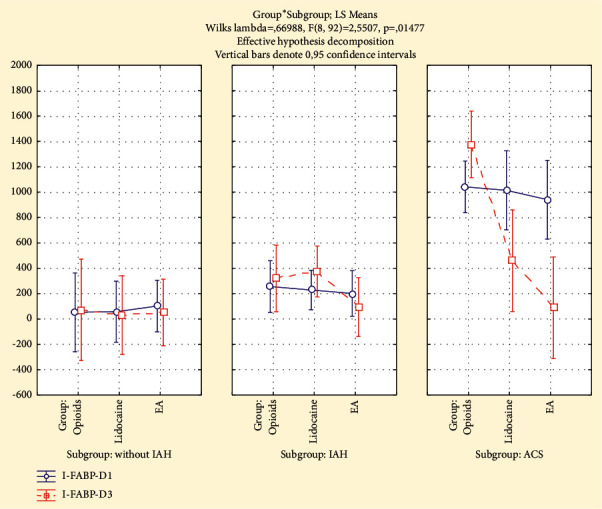
The plasma I-FABP (pg/ml) on day 1 (IAP-D1) and day 3 (IAP-D3) in analgesia groups and subgroups (without IAH, IAH, and ACS).

In children with ACS, the plasma citrulline levels on the third day (D3) were significantly higher than those before the onset of analgesia (D1); they increased in the “Lidocaine” group to 59% (*p*=0.05) and in the “EA” group to 134% (*p* < 0.001). But in the “Opioids” group, plasma citrulline decreased insignificantly to 30% (*p*=0.48) from the onset of analgesia to day 3 (D3). In children with ACS, in the “EA” group, the plasma citrulline levels on the third day (D3) were higher than those in the “Lidocaine” and “Opioids” groups (*p* < 0.05). In the “Lidocaine” group, the citrulline levels on the third day were significantly higher than those in the “Opioids” group (*p* < 0.05). (Figure 5)

### 3.3. The Dynamics of Plasma I-FABP

Among the patients without IAH, the plasma I-FABP increased in the “Opioids” group to 96% from D1 to D3, but it decreased in the “Lidocaine” group to 40% and in the “EA” group to 24% compared to initial levels. In subgroup of patients without IAH, plasma I-FABP levels in the “Lidocaine” and “EA” groups on D3 were significantly lower than those in the “Opioids” group (*p* < 0.05).

The average plasma I-FABP levels among the patients with IAH on postoperative day 3 (D3) were higher (37%) (*p*=0.57) than initial levels on day 1 (D1) in the “Opioids” group, and they were lower than initial levels in the “Lidocaine” (12%) (*p*=0.86) and “EA” (75%) (*p*=0.01) groups. Also, in the “EA” group, on the third day (D3), the plasma I-FABP level was significantly lower than those in the “Lidocaine” and “Opioids” groups (*p* < 0.05).

The plasma I-FABP levels among the patients with ACS decreased significantly during observation period in the “Lidocaine” group to 42% (*p*=0.05) and in the “EA” group to 96% (*p*=0.003), but it increased in the “Opioids” group to 63% (*p*=0.22). In children with ACS, in the “EA” group, the plasma I-FABP levels on the third day (D3) were significantly lower than those in the “Lidocaine” and “Opioids” groups (*p* < 0.05). In the “Lidocaine” group, on the third day, the I-FABP levels were significantly lower than those in the “Opioids” group (*p* < 0.05).

## 4. Discussion

The IAP rises due to a mismatch between the volume of the abdominal cavity and its contents. The enlarged contents of the abdominal cavity are initially compensated by the compliance of the abdominal wall, which prevents excessive growth of IAP. With further enlarging of the abdominal content, IAP rises progressively. Being located directly in the abdominal cavity, the gastrointestinal tract is affected the first and plays a key role in the pathogenesis of IAH. Increased IAP compresses the vessels of the abdominal cavity and microvascular bed, leading to splanchnic hypoperfusion, intestinal ischemia, and necrosis of enterocytes. All these factors result in disruption of the barrier function of the intestinal wall, bacterial translocation and release of proinflammatory mediators in the systemic blood flow, and subsequent development of sepsis and multiple organ dysfunctions. Affected lymph outflow and inflammatory changes of the intestinal wall lead to intestinal edema and further increase in IAP, resulting in the “vicious circle.” The IAH and ACS are independent predictors of mortality among critically ill children and adults. The IAP is detected by measurement of the pressure in urinary bladder. This method is suggested as the “gold” standard for the diagnosis of IAH and ACS and is recommended in all critically ill patients [5].

Scientific literature data suggest that splanchnic ischemia of various origins leads to increased levels of I-FABP and decreased levels of citrulline in the blood due to enterocyte damage and impaired intestinal barrier function [4, 6–8].

Citrulline is an amino acid produced from glutamine in the mitochondria of mature enterocytes in the upper part of the intestinal villi. There is scientific evidence in literature suggesting the decrease in plasma citrulline concentration below 10 nmol/ml as a marker of the decreased enterocyte mass [4]. In our study, the average plasma citrulline concentration was 25.24 nmol/ml in patients without IAH, 9.18 nmol/ml in patients with IAH, and 7.42 nmol/ml in patients with ACS.

Intestinal fatty acid-binding protein (I-FABP) is normally present in the cytoplasm of enterocytes and is not detected in plasma and urine. Concentrations of I-FABP in plasma or urine above 100 pg/ml indicate the enterocyte necrosis [4]. We found the following average levels of plasma I-FABP: 39.54 pg/ml in children without IAH, 148.39 pg/ml in children with IAH, and 868.35 pg/ml in children with ACS.

There are limited data in the literature about correlation between the plasma I-FABP and IAP levels. In the prospective observational study by Strang et al. [9], among 198 severely ill adults, including 60% of subjects with IAH and 8% with ACS, the level of I-FABP in urine was positively correlated with the level of IAP. But the authors did not find the correlation between the serum I-FABP and the IAP. In this study, serum and urinary I-FABP levels did not differ significantly between patient groups with IAH and without IAH. However, the patient group with ACS had significantly higher (*p*=0.037) urine I-FABP levels (235 [85; 1747] mcg/g) compared to the patient group without ACS (87 [33; 246] mcg/g). Based on the data obtained, according to the authors, the I-FABP indicator did not show diagnostic value for the early detection of complications of IAH, including splanchnic ischemia.

In contrast to the abovementioned study by Strang et al. [9], our study demonstrated a statistically significant positive correlation between the plasma I-FABP levels and the IAP values in children with appendicular peritonitis. We did not determine the urine I-FABP. Also, in contrast to the study by Strang et al., in our study, we observed the statistically significant difference (*p* < 0.0001) in the plasma I-FABP between children without IAH (39.54 [31.2; 78.4] pg/ml) and those with IAH (148, 39 [104.67; 262.67] pg/ml). Our findings on significantly higher (*p* < 0.0001) levels of I-FABP in patients with ACS (868.35 [500.8; 1541.5] pg/ml) compared to patients without ACS (148.39 [104.67; 262.67] pg/ml) are concordant with the results demonstrated by Strang et al.

It can be assumed that the differences in the results of our study and the abovementioned study by Strang et al. can be explained by the development of splanchnic hypoperfusion in children at lower IAP levels than in adults, which led to enterocyte damage and I-FABP release.

We did not find scientific literature data on the dependence of plasma citrulline levels on the value of IAP.

Although the optimal method of analgesia in IAH has not been established, some scientific data suggest that the method of analgesia may directly affect the pathogenetic links in the development of IAH. The systemic opioid analgesia may contribute to the development of intestinal paresis through the activation of *μ*-opioid receptors in the gastrointestinal tract and may therefore contribute to rising IAP and presumably causing splanchnic ischemia [10].

No data have been published concerning the use of intravenous lidocaine analgesia in patients with IAH. However, the results of some clinical studies suggest that the intravenous lidocaine analgesia after abdominal surgery has contributed not only to reducing the intensity of postoperative pain but also to faster recovery of intestinal motility associated with suppression of visceral sympathetic pulsation and direct inhibitory effect of lidocaine on the reflexes from the intermuscular plexus (Auerbach). All these can result in reducing the postoperative need in opioids and decreasing in IAP, thus avoiding the development of intestinal ischemia [11].

The administration of thoracic epidural analgesia in patients with IAH was accompanied by a decrease in IAP, which was primarily associated with improved compliance with the abdominal wall, as well as the restoration of peristalsis and reduced extravasation into the third space [12].

Some clinical and experimental data suggest that epidural analgesia improves splanchnic blood flow, thus reducing the risk of visceral ischemia [13, 14].

We could not find any scientific literature data on the influence of postoperative analgesia technique on plasma citrulline and I-FABP levels. In our study, we observed higher levels of citrulline and lower levels of I-FABP in plasma on the third postoperative day in children treated with intravenous infusion of lidocaine and epidural analgesia, compared to patients having intravenous morphine. This is probably due to higher IAP levels in children in opioid group, as well as improved splanchnic blood flow in epidural group.

### 4.1. Limitations

The small number of patients and the fact that this work is a single-center study are both limitations of the study. Additionally, the population was only those with appendicular peritonitis, and the results cannot be applied to patients with IAH or ACS of other etiologies.

## 5. Conclusions

The intra-abdominal pressure had strong inverse correlation with the plasma citrulline level and strong positive correlation with the plasma I-FABP in children operated for appendicular peritonitis.

Patients without intra-abdominal hypertension presented significantly higher plasma citrulline and lower I-FABP levels compared with patients with intra-abdominal hypertension and abdominal compartment syndrome.

Epidural analgesia is highly protective for intestinal wall barrier function in patients at risk of developing intra-abdominal hypertension and abdominal compartment syndrome. Intravenous infusion of lidocaine can be used as an alternative to epidural analgesia.

## Figures and Tables

**Table 1:** The anthropometric and demographic data of patients in analgesia groups.

Data/group	Opioids (*n* = 25)	Lidocaine (*n* = 23)	ЕА (*n* = 26)	*p*
Sex (male/female), *n*	11/14	10/13	10/16	>0.05
Age, months	89 (65–113)	96 (72–119)	115 (87–142)	
Body mass, kg	24 (19–30)	32 (23–41)	36 (28–44)	
Height, cm	125 (114–137)	130 (118–143)	140 (127–153)	

**Table 2:** The surgery types in patients in analgesia groups.

Surgery type/group	Opioids (*n* = 25)	Lidocaine (*n* = 23)	ЕА (*n* = 26)	*p*
Laparotomy by McBurney	13	14	14	>0.05
Median laparotomy	12	9	12	
Appendectomy	25	23	26	
Partial resection of omentum	5	5	6	
Lavage and drainage of abdominal cavity	25	23	26	

**Table 3:** The average pain intensity at rest and during movements and morphine dose in analgesia groups (mean (95% CI)).

Data/group	Opioids (*n* = 25)	Lidocaine (*n* = 23)	ЕА (*n* = 26)
NRS/FLACC at rest, points	1 (1–2)	0 (0–0)	0 (0–0.5)
NRS/FLACC during movements, points	2 (2–3)	1 (0–2)	1 (0–1)
Morphine dose, mcg/kg/24 h	315.35 (187.2–510.3)	0 (0–0)	0 (0–0)

**Table 4:** The average plasma citrulline and I-FABP levels in patients without IAH, with IAH, and with ACS on day 1 (mean (95% CI)).

	Without IAH (*n* = 33)	IAH (*n* = 27)	ACS (*n* = 14)
Citrulline D1, nmol/ml	23.7 (16.0–31.3)	9.3 (7.3–11.3)	6.9 (5.3–8.6)
I-FABP D1, pg/ml	76.9 (32.6–121.1)	226.0 (161.8–290.3)	1011.7 (731.9–1291.5)
IAP-D1, mmHg	7.6 (6.5–8.6)	11.6 (11.2–12.0)	14.7 (12.9–16.6)

## Data Availability

The data used to support this study are available from the corresponding author upon request.

## Abbreviations

IAP: Intra-abdominal pressure
IAH: Intra-abdominal hypertension
ACS: Abdominal compartment syndrome
I-FABP: Intestinal fatty acid-binding protein.
